# Supplementary material for: The design and reconstructible history of the Mayan eclipse table of the Dresden Codex
Source: Sci Adv. 2025 Oct 22;11(43):eadt9039. doi: 10.1126/sciadv.adt9039 (PMC13141895; doi:10.1126/sciadv.adt9039)
Supplement: Supplementary file 1 — Supplementary Text Legends for tables S1 and S2 [file sciadv.adt9039_sm.pdf]

Science Advances
AAAS

Supplementary Materials for

The design and reconstructible history of the Mayan eclipse table of the Dresden Codex

John Justeson and Justin Lowry

Corresponding author: John Justeson, justeson@gmail.com; Justin Lowry, jlowr002@plattsburgh.edu

Sci. Adv. **11**, eadt9039 (2025)
DOI: 10.1126/sciadv.adt9039

The PDF file includes:

Supplementary Text
Legends for tables S1 and S2

Other Supplementary Material for this manuscript includes the following:

Tables S1 and S2

Supplementary Text

Distribution of day lengths for spans of 1 to 405 months

This section provides complete data on the percentage of occurrences of each span in days for every interval of *n* months, $1 \leq n \leq 405$, among first appearances of the lunar crescent at Monte Alban, Oaxaca, Mexico between 650 BCE and 0 BCE. The interval data was compiled by Justeson from Lowry's determination of the times and dates of first appearances of the lunar crescent after new moon, using the Horizons Online Ephemeris System of NASA's Jet Propulsion Laboratory, <https://ssd.jpl.nasa.gov/horizons/>, produced by Jon D Giorgini and the Solar System Dynamics Group.

Lowry determined the times and dates of the first appearance of the lunar crescent after new moon using data from figure 4 of John A. R. Caldwell and C. David Laney 2001 article, "First visibility of the lunar crescent", *African Skies* 5(5)15-23. We determined that the inequality

$$lunar\ elevation > 1.67 \times \sin\left(\frac{\triangle\ azimuth \times \pi}{21}\right) + 3.55$$

distinguishes the 209 examples that they evaluated as observable from all others, under clear viewing conditions, to within a fraction of a degree. Lowry applied this criterion at ten-minute intervals, interpolating to a solar elevation 4° below the horizon.

Table S1. Frequency distribution of the number of days in *n* months for 1 to 405 months.

Table S2: Table of dates for the first visible crescent moon from 650 BCE to 0 BCE, when viewed from the site of Monte Alban.
