# Supplementary material for: The design and reconstructible history of the Mayan eclipse table of the Dresden Codex
Source: Sci Adv. 2025 Oct 22;11(43):eadt9039. doi: 10.1126/sciadv.adt9039 (PMC13141895; doi:10.1126/sciadv.adt9039)
Supplement: Supplementary file 2 [file sciadv.adt9039.v1.pdf]

ANTHROPOLOGY

The design and reconstructible history of the Mayan eclipse table of the Dresden Codex

John Justeson[1]* and Justin Lowry[2]*

Copyright © 2025 The Authors, some rights reserved; exclusive licensee American Association for the Advancement of Science. No claim to original U.S. Government Works. Distributed under a Creative Commons Attribution License 4.0 (CC BY).

This paper explores how ancient Mayan calendar specialists designed a predictive eclipse table, revising a century of interpretation. The table's length, 405 months, was originally implemented in a general lunar calendar table of 405 successive months; within a few passes through this lunar calendar, intervals among observed eclipses could have stimulated an approximation to the series of lunar intervals that were later compiled as stations of an eclipse table; and for all and only these lunar stations to correspond to dates of upcoming eclipses, dates in successive 405-month eclipse tables would have to have overlapped. It identifies optimal procedures for the amounts of overlap that would maintain its predictions' correctness and shows that these procedures could yield a sequence of tables that would anticipate every solar eclipse observable in the Mayan territory from a century or two after the first evidence of the Mayan lunar calendar to at least the era of the extant eclipse table, 700 years later.

INTRODUCTION

More than 2000 years before the European invasion of the Americas, indigenous civilizations of Mexico and Guatemala had developed and used both a "divinatory" calendar of 260 days and a civil calendar of exactly 365 days. The divinatory calendar is so called because it was used by calendar specialists to divine the fates of individuals on the basis of the date of their birth in that calendar. By approximately 500 BCE, it had become associated with lunar phenomena. It associated the calendar name of a night on which the Moon was visible with the number of nights it had been visible since the last night of its invisibility, at new moon. The divinatory calendar was also applied to dates within planetary cycles, whose earliest surviving records are first attested centuries later.

As Steele (*1*) has stated, "One of the most awe-inspiring of celestial events seen by early man must surely have been the occurrence of an eclipse of the Sun or Moon. With no apparent warning, one or two times a year, a darkness encroaches upon the bright light of the Sun or Moon; sometimes to completely cover the heavenly body, sometimes to retreat before the light is fully extinguished … [and] in the rare event of a total solar eclipse, the day may literally turn into night during which stars become visible and the air turns cold. It is therefore expected that eclipses were viewed as important astrological events in many early civilizations.

Unfortunately, however, our understanding of Mayan astronomy is severely hindered by the relatively small amount of material available for study… One of the surviving works, now known as the Dresden Codex, contains a table for predicting eclipses, but its exact workings are not yet fully understood."

This paper details the Mayan development of eclipse prediction, starting from what is now known about the work of Mayan calendar specialists ("daykeepers"). Key to this development was their regular tracking of days within lunar months, counted from the first appearance of the lunar crescent after new moon, and such long-term calendrical calculations as survive in their workplaces. It addresses how daykeepers developed a model for eclipse prediction from a more general lunar month model and how daykeepers' understanding of the short-term and long-term timing of solar eclipses observable in the Mayan territory developed from these beginnings. Together, this yields a more detailed account of daykeepers' model for the timing of solar eclipses observable in the Mayan territory that are reflected in the eclipse table of the Dresden Codex.

[1]University at Albany, Albany, NY, USA. [2]SUNY, Plattsburgh, NY, USA.
*Corresponding author. Email: justeson@gmail.com (J.J.); jlowr002@plattsburgh.edu (J.L.)

RESULTS

The eclipse table of the Dresden Codex consists of 69 new moon dates within an overall span of 405 new moons from the base date of the table to its final date. Fifty-five of these dates were intended to anticipate dates on which a solar eclipse might take place, within a series of six or (once) seven eclipse-possible dates that occur at successive intervals of six lunations. The other 14 dates involve spans of 11 or 17 lunations that separate one eclipse-possible date from the next, a result of the accumulating difference between the span of six new moon appearances, averaging about 177.1835 days, and the nodes of the eclipse cycle, averaging 173.30906 days.

Previous work with the eclipse table has consistently assumed that a successor table of the same structure would be calculated from a base that was the last station of the preceding table. However, its length of 405 months was not designed for eclipse prediction; unanticipated eclipses could occur in the application of the next table or two if the final station of one table was used as the base for composing the next, and increasingly with each successive resetting.

Applications of the 11,960-day to 405-month ratio in Mayan hieroglyphic texts are almost consistently interpreted as eclipse based, being the length of the eclipse table. Lounsbury's 1447-day model (see the "Modeling the length of the lunar month" section below) suggests an alternative: that it was applied by Mayan daykeepers to compute month intervals in general, likely implemented in a table of day lengths for 1 to 405 lunar months, which was only later adapted for use in eclipse tables.

Under a 1447-day lunar model, months 44 and 88 span 1299 (= $[21 \times 29] + [23 \times 30]$) and 2599 (= $[41 \times 29] + [47 \times 30]$) days, equaling $[260 \times 5] - 1$ and $[260 \times 10] - 1$ days, and months 317 and 361 fall on days 9361 (= $[149 \times 29] + [168 \times 30]$) and 10,661 (= $[169 \times 29] + [192 \times 30]$), equaling $[36 \times 260] + 1$ days. These intervals are the only month spans among the first 404 to fall within 1 day of a multiple of 260 days. In all three arrangements of the 17- and

15-month intervals, month 405 (= 44 + 361 and 88 + 317) falls on day 11,960 = 1299 + 10,661 = 2599 + 9361; it is the first month span to fall at a multiple of 260 days in a 1447-day lunar model.

This is not plausibly a coincidence. Four hundred five months approximate a lunar month cycle much more closely than an eclipse cycle: The average length of 405 lunations falls just 0.11259 days short of 11,960 days, while 69 nodal passages average 1.67486 days short of it—about 15 times larger. Furthermore, in each of the eight uninterrupted series of the eclipse table's six or (once) seven intended stations, 6 months apart, one station averages closer to a node than does 11,960 days; these stations occur at 1388, 2599, 3987, 5198, 6586, 7973, 9184, and 10,572 days. All this suggests that daykeepers had initially designed the 405-month interval as a commensuration of the lunar cycle with the divinatory calendar in a sequence of 405 successive months—whether or not Mayan daykeepers, or their predecessors, had used a ratio of 1447 days to 49 months as a computing model for lengths of month sequences.

Instead, this paper shows that new eclipse tables were most likely started at one of two earlier stations in the table: at 358 lunations (its average offset from 61 intereclipse intervals being 0.0971 days shorter) or at 223 lunations (its average offset from 38 intereclipse intervals being 0.4236 days longer). With a mix of four resettings at 385 months for each resetting at 223 months, the calendrical interval is [4 × 10,592] + 6585 = 48,873 days, while [4 × 358] + 223 = 1655 lunations average 48,873.15 days. Restarting an eclipse table at these intervals, and at these ratios, would have enabled daykeepers to reset the table reliably for a few millennia.

DISCUSSION

Floyd Lounsbury's framework

Lounsbury (*2*) treated a wide range of issues concerning the eclipse table within a framework based on "three items of information that can be derived from naked-eye observations and record keeping with a calendar, or inferred from an accumulation of such records, namely, the average length of the lunar month, the average length of the interval between lunar nodes, and the ecliptic limit for solar eclipses." The key for all subsequent work is his determination of the day on which each of the eclipse table's stations fell in the Mayan 260-day divinatory calendar based on a systematic procedure for identifying the number of days separating each from the eclipse table's base, "from three separate kinds of data contained in it ... (a) the series of intervals...; (b) the series of cumulative totals...; and (c) ... the days in the 260-day almanac on which the respective eclipse possibilities may fall." This was necessary because, for some stations, one of the table's three recorded dates is inconsistent with the other two. Figure 1 presents Lounsbury's corrections of these inconsistencies; for each station, the number of days from the base of the table appears in column 5. The final, unnumbered columns give Justeson's (*3*) categorization of 55 stations as "real," intended by its users as dates on which a solar eclipse might be observed, and 14 as "contrived," lying too far from the nodes of the eclipse cycle for a solar eclipse to be observed, but included in the table so that successive stations were always 6 or, in nine cases, 5 months apart.

Lounsbury's observations are applied here to draw more detailed inferences about the eclipse table's structural features, about features of a prior table that he demonstrated must have existed and been modified during the creation of the Dresden version, and about procedures by which successive tables were compiled. This elaboration is anchored in Lounsbury's proposed Mayan model for the length of a lunation and its multiples, in structural features of the table worked out by Justeson (*3*), and crucially by commensurating dates of observed eclipses with the 260-day calendar. Twenty-one different historical sequences of tables prove to be consistent with these features, with 16 viable from before the earliest attestation of a Mayan lunar calendar.

Lounsbury (*2*) used a 1-month range around the nodes—points along the plane of Earth's orbit around the Sun when Earth, the Sun, and the Moon are aligned and a solar or lunar eclipse can occur—as an exploratory device for the range of eclipse-possible dates across the length of the table. That range, however, was too broad. In the Mayan territory, the maximum abnodal range for total solar eclipses is 23 calendar days, i.e., as long as about $23^1/_2$ days between a sunrise eclipse and, years later, a sunset eclipse. In the Maya lowlands, the longest interval between sunrise and sunset is 13 hours and 13 min, around summer solstice (*4*), so the maximum abnodal interval there can be estimated at 23.55069 days. This paper therefore pursues the Mayan development of models for warning of possible eclipses within ±11.775 (≈ 23.55069/2) days of nodal passage.

Eclipses visible in Mayan territory, 350 to 1150 CE

Bradley Schaefer determined that a solar eclipse is noticeable near a horizon with only about 25% of the solar disc covered (*3*) but only with about 94% coverage at the zenith. Absent a verified quantitative representation of observability in terms of altitude and coverage, this paper implements Schaefer's characterization by the linear inequality

$$\text{Magnitude} \geq (\text{Altitude} - 0.25)/(0.94 - 0.25) \qquad (1)$$

Justeson used Jubier's online application (*5*) to determine each solar eclipse's maximum magnitude and corresponding altitude across the Mayan territory. One hundred forty-five solar eclipse dates satisfy this criterion (see Fig. 2). All are varieties of total eclipse—total, annular (when the Moon is close enough to Earth that it does not completely cover the solar disk), or hybrid (varying over its path between total and annular); the central path of partial solar eclipses is usually far from the equator, rarely with any coverage of the solar disk in Mesoamerica. (Surrogate methods for verifying eclipses, such as viewing images of the eclipsing Sun projected onto the ground through tree leaves, are not entertained in this study, because it is plausible, even likely, that regular practices of this sort presuppose an ability to anticipate when a solar eclipse might occur.)

The earliest currently known lunar day count in a Mayan text is the partially preserved example on Naachtun Stela 23 (*6*, *7*), dated 1 August 361 CE. It appears on the earliest dated monument of the leaders of a Teotihuacan-associated takeover of the central Peten, Guatemala (*8*), after expanding southward following their takeover of parts of southern Veracruz by around 300 CE (*9*). The Fig. 2 sample begins with the eclipse of 356 CE, when the invaders were likely active in the Peten, just before they began ruling there; it ends with the last eclipse before the latest plausible placement of the eclipse table's last station in 1148 CE (*3*). All these eclipses were total, annular, or hybrid—i.e., none is partial or penumbral. Those from 356 to 900 CE were observable throughout the Classic Mayan territory; those after 900 CE were observable in northern Yucatan at major

Dresden Codex page number	Column number (= node number)	Cumulative totals as written in the Codex	Corrections required by indicated almanac days	Day name in 260-day calendar and its position in a 520-day cycle	Day from the lower line of almanac days (with its number in the almanac)	Increment	Intended	*Contrived*	Intended	*Contrived*
					13 Muluc (169)					
53a	1	7.17	1.0	8.17 177 [6]	8 Cimi (346)	177	**177**		**177**	
	2	17.13	1	17.14 354 [12]	3 Akbal (3)	177	**354**		**177**	
	3	1.7.2		1.7.2 502 [17]	8 Chuen (151)	148		*502*		
	PICTURE									
	4	1.15.14	5	1.15.19 679 [23]	3 Lamat (328)	177		*679*		
	5	2.6.16		2.6.16 856 [29]	11 Chicchan (505)	177	**856**			*502*
	6	2.15.13		2.15.13 1,033 [35]	6 Ik (162)	177	**1033**		**177**	
54a	7	3.6.11		3.6.11 1,211 [41]	2 Ahau (340)	178	**1211**		**178**	
	8	3.15.8		3.15.8 1,388 [47]	10 Caban (517)	177	**1388**		**177**	
	9	4.6.5		4.6.5 1,565 [53]	5 Ix (174)	177	**1565**		**177**	
	10	4.15.8	−6	4.15.2 1,742 [59]	13 Chuen (351)	177	**1742**		**177**	
	11	5.5.19		5.5.19 1,919 [65]	8 Lamat (8)	177		*1919*		
	12	5.10.16	4.0	5.14.16 2,096 [71]	3 Chicchan (185)	177		*2096*		
	13	6.3.4	1.0	6.4.4 2,244 [76]	8 Ben (333)	148	**2244**			*502*
55a	PICTURE									
	14	8.13.2	−2.0.0	6.13.2 2,422 [82]	4 Chuen (511)	178	**2422**		**178**	
	15	7.3.18	1	7.3.19 2,599 [88]	12 Lamat (168)	177	**2599**		**177**	
	16	7.12.16		7.12.16 2,776 [94]	7 Chicchan (345)	177	**2776**		**177**	
	17	8.3.13		8.3.13 2,953 [100]	2 Ik (2)	177	**2953**		**177**	
	18	8.12.10		8.12.10 3,130 [106]	10 Cauac (179)	177	**3130**		**177**	
56a	19	9.1.18		9.1.18 3,278 [111]	2 Manik (327)	147		*3278*		
	PICTURE									
	20	9.10.15		9.10.15 3,455 [117]	10 Kan (504)	177	**3455**			*325*
	21	10.1.12		10.1.12 3,632 [123]	3 Imix (161)	177	**3632**			
	22	10.10.9		10.10.9 3,809 [129]	13 Etznab (338)	177	**3809**		**177**	
57a	23	11.1.6	1	11.1.7 3,987 [135]	9 Cib (516)	178	**3987**		**178**	
	24	11.10.4		11.10.4 4,164 [141]	4 Ben (173)	177	**4164**		**177**	
	25	12.1.0	1	12.1.1 4,341 [147]	12 Oc (350)	177	**4341**		**177**	
	26	12.8.8	1	12.8.9 4,489 [152]	4 Etznab (498)	148		*4489*		
	PICTURE									
58a	27	12.17.5	1	12.17.6 4,666 [158]	12 Men (155)	177		*4666*		
	28	13.8.2	1	13.8.3 4,843 [164]	7 Eb (332)	177	**4843**			*502*
	29	13.17.0	1	13.17.1 5,021 [170]	3 Oc (510)	178	**5021**		**178**	
	30	14.7.17	1	14.7.18 5,198 [176]	11 Manik (167)	177	**5198**		**177**	
51b	31	14.16.14	1	14.16.15 5,375 [182]	6 Kan (344)	177	**5375**		**177**	
	32	15.7.11	1	15.7.12 5,552 [188]	1 Imix (1)	177	**5552**		**177**	
	33	15.16.8	1	15.16.9 5,729 [194]	9 Etznab (178)	177	**5729**		**177**	
	34	16.7.5	1	16.7.6 5,906 [200]	4 Men (355)	177		*5906*		
	35	16.16.2	1	16.16.3 6,083 [206]	12 Eb (12)	177		*6083*		
	36	17.5.10	1	17.5.11 6,231 [211]	4 *Ahau (160)	148	**6231**			*502*
52b	PICTURE									
	37	17.14.8	1	17.14.9 6,409 [217]	13 Etznab (338)	178	**6409**		**178**	
	38	18.5.5	1	18.5.6 6,586 [223]	8 Men (515)	177	**6586**		**177**	
	39	18.14.2	1	18.143 6,763 [229]	3 Eb (172)	177	**6763**		**177**	
	40	19.4.19	1	19.5.0 6,940 [235]	11 Muluc (349)	177	**6940**		**177**	
53b	41	19.13.16	1	19.13.17 7,117 [241]	6 Cimi (6)	177	**7117**		**177**	
	42	1.0.3.4	1	1.0.3.5 7,265 [246]	11 Ix (154)	148		*7265*		
	PICTURE									
	43	1.0.12.1	1	1.0.12.1 7,442 [252]	6 Chuen (331)	178	**7442**			*325*
	44	1.1.2.18	1	1.1.2.19 7,619 [258]	1 Lamat (508)	177	**7619**		**177**	
	45	1.1.11.15	1	1.1.11.16 7,796 [264]	9 Chicchan (165)	177	**7796**		**177**	
54b	46	1.2.2.12	1	1.2.2.13 7,973 [270]	4 Ik (342)	177	**7973**		**177**	
	47	1.2.11.9	1	1.2.11.10 8,150 [276]	12 Cauac (519)	177	**8150**		**177**	
	48	1.3.2.6	1	1.3.2.7 8,327 [282]	7 Cib (176)	177	**8327**		**177**	
	49	1.3.9.14	1	1.3.9.16 8,475 [287]	12 Kan (324)	148		*8475*		
	PICTURE									
	50	1.4.0.11	1	1.4.0.12 8,652 [293]	7 Imix (501)	177		*8652*		
55b	51	1.4.9.8	1	1.4.9.9 8,829 [299]	2 Etznab (158)	177	**8829**			*502*
	52	1.5.0.6	1	1.5.0.7 9,007 [305]	11 Cib (336)	178	**9007**		**178**	
	53	1.5.9.3	1	1.5.9.4 9,184 [311]	6 Ben (513)	177	**9184**		**177**	
	54	1.6.0.0	1	1.6.0.1 9,361 [317]	1 Oc (170)	177	**9361**		**177**	
	55	1.6.8.17	1	1.6.8.18 9,538 [323]	9 Manik (347)	177	**9538**		**177**	
	56	1.6.17.14	1	1.6.17.15 9,715 [329]	4 Kan (4)	177	**9715**		**177**	
	57	1.7.8.11	1	1.7.8.12 9,892 [335]	12 Imix (181)	177		*9892*		
	58	1.7.15.19	1	1.7.16.0 10,040 [340]	4 Muluc (329)	148	**10040**			*325*
	PICTURE									
	59	1.8.6.16	1	1.8.6.17 10,217 [346]	12 Cimi (506)	177	**10217**		**177**	
	60	1.8.15.14	1	1.8.15.15 10,395 [352]	8 Kan (164)	178	**10395**		**178**	
	61	1.9.6.11	1	1.9.6.12 10,572 [358]	3 Imix (341)	177	**10572**		**177**	
	62	1.9.15.8	1	1.9.15.9 10,749 [364]	11 Etznab (518)	177	**10749**		**177**	
57b	63	1.10.6.5	1	1.10.6.6 10,926 [370]	6 Men (175)	177	**10926**		**177**	
	64	1.10.15.2	1	1.10.15.3 11,103 [376]	1 Eb (352)	177	**11103**		**177**	
	65	1.11.4.10	1	1.11.4.11 11,251 [381]	6 Ahau (500)	148		*11251*		
	PICTURE									
	66	1.11.13.7	1	1.11.13.8 11,428 [387]	1 Caban (157)	177	**11428**			*325*
	67	1.12.4.4	1	1.12.4.5 11,605 [393]	9 Ix (334)	177	**11605**		**177**	
58b	68	1.12.13.1	1	1.12.13.2 11,782 [399]	4 Chuen (511)	177	**11782**		**177**	
	69	1.13.3.18	1	1.13.3.19 11,959 [405]	12 Lamat (168)	177	**11959**		**177**	

Fig. 1. Eclipse table stations and categorization. Lounsbury's (*2*) determination of the eclipse table's stations, with Justeson's (*3*) categorization of each as "intended" (observable locally) or "contrived" (not observable locally), fitting a purely formal model. A horizontal band separates entries along the top half of the pages, conventionally numbered 53a through 58a; they are read in sequence from left to right. Pages 53b to 58b, along the bottom of the same pages, are read afterward. The placements of the iconographic elements seen in Fig. 6 are displayed in this figure and marked by the word "PICTURE."

**356** May 16	**568** Dec 3	**758** Apr 12	**931** Jun 18
**363** Jun 27	**577** Jul 1	**765** Nov 17	**934** Apr 16
**364** Jun 16	**580** Apr 29	**771** Jan 20	**941** Nov 21
**385** Oct 20	**580** Oct 24	**772** Jan 9	**964** Mar 16
**396** Mar 25	**584** Aug 11	**778** Aug 26	**968** Jan 1
**396** Sep 18	**587** Dec 4	**783** Jun 3	**981** Sep 30
**399** Jan 23	**592** Sep 11	**790** Jul 16	**985** Jul 20
**404** Apr 25	**608** Oct 14	**793** Nov 8	**995** Dec 24
**411** Nov 30	**619** Mar 21	**797** Aug 26	**997** Jun 7
**413** Apr 16	**623** Jan 6	**783** Jun 3	**1003** Jul 31
**420** Nov 21	**624** Jun 21	**790** Jul 16	**1010** Mar 18
**424** Sep 9	**630** Aug 13	**793** Nov 8	**1011** Aug 31
**428** Jun 28	**631** Aug 2	**798** Feb 20	**1022** Feb 2
**436** Jul 29	**634** Jun 1	**804** Apr 13	**1025** May 29
**439** May 28	**635** Nov 15	**805** Sep 26	**1035** Nov 1
**439** Nov 22	**638** Sep 13	**815** Mar 14	**1043** Jun 9
**447** Jan 1	**642** Jul 1	**826** Feb 10	**1048** Sep 10
**450** Apr 27	**650** Feb 6	**828** Dec 10	**1050** Jan 25
**450** Oct 21	**652** Jun 11	**830** May 25	**1061** Dec 14
**453** Feb 24	**662** Nov 16	**837** Jul 6	**1062** Dec 2
**474** Dec 24	**664** May 1	**847** Dec 11	**1065** Oct 2
**490** Aug 31	**689** Dec 17	**851** Sep 28	**1076** Mar 7
**493** Jun 29	**696** Feb 8	**852** Mar 24	**1077** Feb 25
**500** Aug 10	**696** Aug 2	**855** Jul 17	**1079** Jul 1
**502** Jan 24	**704** Mar 10	**856** Jan 11	**1083** Apr 19
**507** Mar 29	**707** Jan 8	**859** Oct 29	**1090** May 31
**511** Jan 15	**710** May 2	**877** May 16	**1094** Sep 12
**515** Apr 29	**711** Oct 16	**880** Mar 25	**1097** Jul 11
**526** Mar 28	**716** Dec 18	**881** Aug 28	**1102** Oct 13
**530** Jul 10	**728** May 13	**884** Jun 26	**1105** Aug 11
**533** Nov 2	**736** Jun 13	**892** Jul 27	**1116** Jan 16
**537** Aug 21	**739** Apr 12	**899** Sep 8	**1123** Feb 27
**552** May 9	**743** Jul 25	**902** Jan 12	**1130** Apr 9
**554** Sep 12	**744** Jan 19	**905** Oct 30	**1131** Mar 30
**558** Jul 1	**750** Mar 12	**906** Oct 20	**1137** May 21
**559** Dec 14	**750** Sep 4	**924** May 6	**1140** Mar 20
**565** Feb 16			

Fig. 2. Dates of solar eclipses judged observable in the Mayan territory. 350 to 1148 CE.

sites such as Uxmal in the west, Cozumel and Tulum along the east coast, and Chich'en Itzaj in between.

There are 10,440 (= 145 × 144/2) individual intervals among these eclipses. The most frequent interval among them is 669 months, a triple-saros cycle (3 × 223 months); it relates 62 pairs among the 145 eclipses, about $42^3/_4$% of eclipses that fall at least 669 months after the 356 CE eclipse that begins the set. Solar eclipses reliably occur 223 months apart but not at the same point on Earth; however, after 669 months, they tend to occur near the same east/west longitude and around the same time of day (*10*, *11*). It would have been the most reliable predictor of solar eclipse observability available to Mesoamerican daykeepers (*3*). However, multiples of 669 months do not perform impressively; the number of eclipses separated by $669n$ months in the sample declines exponentially, as $y = 89.12 \times 10^{-0.448x}$ ($r^2 = 0.9727$), with just one instance at 9 × 699 months and none at higher multiples (10 × 699 through 14 × 699 months) in the chronological range of Fig. 2.

The next most frequent intervals among these eclipses are 42 instances of 493 months and 39 instances of 1162 months. Their abnodal shifts are close to the same magnitude but opposite signs, +0.61934 days for 493 months and −0.64946 days for 1162 months (34 times); the combined interval of 1655 = 493 + 1162 months yields an overall shift of −0.03125 days—the smallest magnitude shift within that span. Twenty-four eclipses in the sample occur 1655 months after an earlier one, about 19.35% of those at least 1655 months after the first; 47 other pairs are separated by a multiple of 1655 months. The suitability of these intervals for projecting eclipse-possible dates would likely have been recognized over time. Intereclipse intervals of 493 months, with 42 instances across the sample, are more frequent than for any shorter interval, and they occur within each of the first 16 of the sample's 19 493-month intervals, between 356 and 994 CE. The sample spans at most seven multiples of 1162 months; every multiple is instantiated.

From a predictively reliable solar eclipse table, a table with every station 1655 months later proves to be equally viable (see the "Composing a successor eclipse table" section below); the analysis can therefore proceed on the basis of spans of 1655 months or less. Three thousand two hundred pairs of eclipses in the sample fall in this range; 320 different intervals occur, averaging 10 instances per interval. Seven hundred forty-seven pairs are at an interval occurring in the eclipse table as an intended station. The month position of each of the 55 intended stations is matched by one of these intervals, whose frequencies range from 28 instances at 176 months to only one at 341 months, averaging 13.58 instances each; ordered by frequency, this set is consistent with a uniform distribution, fitting a linear equation with $r^2 = 0.9839$. Thirty-seven pairs are at intervals agreeing with 8 of the 14 contrived stations; examples range from 1 to 10 examples per station, 4.63 on average. Fifty-four pairs occur on 1 of 13 intervals that do not occur as stations in the eclipse table, 3.92 on average; 30 fall 1 month later than a contrived interval on months 18, 153, 159, 288, 294, and 382; 21 fall 1 month earlier on months 64, 70, 199, 205, 328, 334, and 375, frequencies ranging from one to eight instances; and 3 are offset by a month from an intended station, one each on months 328 and 375, a month before an intended station, and one on month 341, a month after an intended station.

Modeling the length of the lunar month

Lounsbury (*2*) observed that a ratio of 1447 days to 49 months is by far the best short-term Mayan model for the length of a lunar month. His undergraduate training was as a mathematician (*12*); he had a fascination with number theory, with a particular interest in the use of continued fractions to determine the best integer approximations to fractional values. He arrived at the 1447:49 ratio by this method; explicitly, using 29.530589 days for an average lunation

$$\begin{aligned} 1/0.530589 &= 1 + 0.8846979\ldots = 1 + (1/1.1303292\ldots) \\ &= 1 + \{1/[1 + (1/7.6528726\ldots)]\} \end{aligned} \tag{2}$$

given that 0.6528726... is approximately $^2/_3$, this calculation yields the approximation

$$\begin{aligned} 1 + (1/\{1 + [1/(7 + {}^2/_3)]\}) &= 1 + \{1/[1 + 1/({}^{23}/_3)]\} \\ &= 1 + [1/({}^{26}/_3)] = 49/26 \end{aligned} \tag{3}$$

Therefore, 29.530589 ≈ 29 + 26/49 and 49 × 2926/49 = 1447 for an average month length estimate of 1447/49 = 29.530612245 days. This is just 2 s longer than the average month length across the 9695 months between the earliest and latest solar eclipses in Fig. 2, averaging 29.5305651825 days; a ratio of 1447 days to 49 months would be a viable Mayan model for calculating intervals among lunar stations. In Justin Lowry's compilation of 8052 successive lunar

first appearances at Monte Alban, the ancient Zapotec capital (table S2), a series of 82 successive spans of 98 months across 650 years exemplifies this ratio in 89.024% of cases as 2894 days, with only 5.532% 1 day earlier and 5.444% 1 day later (table S1, intervals of 98 months). Satterthwaite (*13*) had previously mentioned this ratio obliquely in a critique of a proposal by Hermann Beyer and suggested occasional adjustments, not obviously correct, to improve it. Its only previous mention seems to be an approximately 1699 proposal by Isaac Newton, recently found among his unpublished manuscripts (*14*).

A 405-month lunar table and the divinatory calendar

Teeple (*15*) explored the 405-month span of the eclipse table and the ratio of 11,960 days to 405 months and the equivalent ratio of 2392 days to 81 months in intervals among recorded lunar day counts at Palenque in a 1930 monograph on Mayan astronomy. He also demonstrated a systematic use of the less accurate ratio of 4400 days to 149 months at Copan (*15*); his data suggest that it was introduced to link a canonical 5 × 360-day station in the Mayan vigesimal "long count" calendar (9.12.10.0.0 in Mayanist notation) to its beginning date, exactly 135 × 4400 days earlier. Cases and colleagues (*6*, *16*) identified additional Mayan ratios, differing from Palenque's and Copan's, at Bonampak, El Cayo, Coba, Naranjo, Calakmul, Yaxchilan, Tikal, and Dos Pilas.

Features of the eclipse table suggest that, for modeling the lengths of a series of lunar months, daykeepers paid special attention to intervals for which one span in days stood out as particularly common. Of the table's 14 17-month intervals between a contrived station and another station, contrived or intended, nine are of 502 days, and five are of 503 days; in contrast, all 13 17-month intervals between two intended stations are 502 days long (*3*). This contrast has a χ^2 of 12.9486; its probability of occurring by chance is 0.00017, about 1 in 5900. For calculating between new moons of interest, such as eclipse-possible dates, daykeepers appear to have favored the most common length in days for at least some spans in months.

If daykeepers pursued such an approach systematically, the single most reliable ratio of days to months would be achieved by sequences of 98 (= 2 × 49) months: Among the 8052 successive lunar first-appearance dates at Monte Alban, from 650 through 0 BCE, 89.024% of 7954 overlapping 98-month intervals were 2894 days long. Eleven intervals shorter than 405 months have more reliable ratios than that of 11,960 days to 405 months—in order of overall frequency, spans of 98, 209, 111, 13, 307, 362, 83, 224, 294, 279, and 15 months (table S1).

Different spans with identical ratios do not perform identically. The ratio of 2894 days to 98 months is identical to Lounsbury's ratio of 1447 days to 49 months. Observationally, the shorter ratio seems unlikely to have been a rationale for its adoption. While 1447 days is the most frequent length of a 49-month interval, among 8004 examples in the 651-year Monte Alban corpus, only 47.082% are of 1447 days, with almost equal numbers a day longer or shorter; four examples (0.050%) are of 1445 days. Similarly, the ratio of 11,960 days to 405 months accounts for 81.627% of 405-month intervals, while the same ratio in Teeple's model of 2392 days for 81 months accounts for only 66.905% of 81-month intervals (table S1, intervals of 81 and 405 months); $(1 - 0.66905)/(1 - 0.81627) \approx 1.8013$, so the 81-month interval has more than 80% more alternative lengths.

In a 1447-day lunar model, placements of stations in a 405-month series repeat exactly after $49 \times 405 \times (1447/49) = 405 \times 1447 = 586,035$ days ≈ 1604½ years, yielding 49 slightly different models for a 405-month series. Results depend on the order among the 17-month groups and the 15-month group in a table's first 49 months; with 49 different starting points for each order, there are 147 distinct models for a 405-month series. The 405-month station is of 11,959 days on the final station after 14 models: in all starting arrangements of the 15th, 25th, and 35th tables; on the 17-15-17 and 17-17-15 starting arrangement of the 5th table; on the 17-15-17 and 15-17-17 starting arrangement of the 45th table; and on the 17-17-15 starting arrangement of the 44th table.

The placements of all 69 stations in the Dresden Codex agree to within 1 day with their placements in all 147 models; however, across all 11,959-day table models, the number of exact agreements ranges from 41 to 45 stations, on average $43\frac{2}{14}$—just more than 62.5%. As the Dresden table is 11,959 days long, the placements of eclipse-possible dates would have to be adapted, if related to the 1447-day lunar model, or determined using a different model. The case for a different eclipse model is straightforward.

Early observations

The earliest clearly pertinent solar eclipses in the Mayan territory are from the era when Teotihuacanoid leaders took control there, as their ascendancy coincides with the introduction of lunar dates in Mayan texts. Figure 3 gives the frequency distribution of intereclipse intervals occurring more than once within 3 × 405 months of the first solar eclipse they would likely have observed there, on 16 May 356 CE. Only three of Fig. 2's eclipses fell in the first 405 months thereafter, yielding $4 \times (4 - 1)/2 = 6$ intervals among them, all distinct. The first eclipse of the next 405 months fell 405 months after that of June 363; if the 405-month commensuration with the divinatory calendar was already in use by then, this would be a thought-provoking interval. By the end of this series of 405 months, a total of 11 eclipses would have been observed, with 55 intereclipse intervals; among them, an interval of 94 months occurred three times and intervals of 100, 111, 205, and 493 (= 88 + 405) months occurred twice. The first 493-month interval took place 405 months after the second eclipse of the series, which had occurred 88 months after the first; imaginably, this might have raised the possibility of a role for the 405-month span in eclipse occurrence.

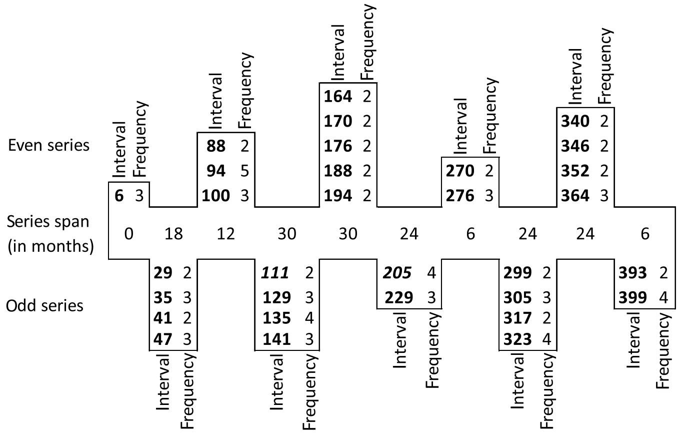

Fig. 3. Frequency distribution of repeated intereclipse intervals among the first 3 × 405 months in the Mayan territory, 356 to 454 CE. The italicized entries for 111 and 205 months correspond to contrived stations of the eclipse table.

After three spans of 405 months, with 190 intereclipse intervals among 20 eclipses, a fairly uniform pattern would emerge (see Fig. 3). Five sets of even intervals alternate with five sets of odd intervals, all but one with multiple stations. Forty intervals occur between pairs of stations within each of the nine sets having multiple intereclipse intervals; within the sets, 16 are 6 months apart, with the rest a multiple of 6 months. The maximum span within a set is 30 months, between 164 and 194 months and between 111 and 141 months. Intervals between successive sets of even and odd groups are regular: The interval between each station in the second of the two groups and each in the earlier group is always 1 month less than a multiple of 6 months, and the smallest span between the last station of a group and the first of the next group is 11 months.

The series from months 164 to 194 includes five intervals at multiples of 6 months apart; a span of 182 months would presumably follow 176 and precede 188 by 6 months for a series of six successive spans of 6 months (one interval of 182 months also occurs in the first 3 × 405 months). A hypothetical approximation to a complete table might begin on an assumption that all complete series might include exactly six successive stations; actually, however, one series in the eclipse table, from months 340 to 376, has seven.

Another series would be nearly complete: Six stations including months 340 to 364 would span 340 to 370 or 334 to 364 months. This series' first station would fall 176 or 182 months after that in the fourth prior series. From the first station of one series of six stations to that of the fourth subsequent series, plus minimally 5 months from the end of each series to the beginning of the next, amounts to only 4 × 30 + 4 × 5 = 140 months. The minimum distance between the first stations of two series that are four sets apart is 176 months, and that between the last stations of two series at four sets apart is 170 months; these minima are achieved between each set spanning 30 months and a subsequent set spanning 24 months. Depending on whether the missing station in the 24-month spans begins or ends the series, the actual range would fall between 164 and 182 months, an excess of 24 to 42 months. Given that six or seven missing 6-month groups therefore intervene between successive stations, plus a 5-month group, the interval between the final station of one group and the first of the next must be at least 11 and at most 17 months.

METHODS

Applying this constraint throughout:

1) The series whose earliest attested interval is 129 months would be expected to begin 30 + either 11 or 17 months before month 164, i.e., beginning on month 117 or 123, spanning months 117 to 147 (the Dresden eclipse table's placement) or 123 to 153.

2) The next prior series would be expected to begin 41 or 47 months earlier on month 94 or 100; this yields months 70 to 100, 76 to 106 (the table's placement), or 82 to 112.

3) The series of three sets earlier would be projected to begin between 90 + 33 and 90 + 51 months earlier to month 41, 35, or 29; the span of 29 months (the table's placement) is attested, suggesting (correctly) month 59 for that set's last station.

4) The end of a series projected four sets earlier would fall on month 12 (the table's placement) or 18. The preceding station would be month 6, attested three times in the 3 × 405-month sample. The beginning of its series would be projected to 18 or 12 months before the table's base. If the base corresponds to month 405 of an immediately prior table, these options are to month 387 (the table's placement) or 393, the latter appearing twice in the sample.

Resolving all these placements would require additional eclipse observation, but the data suggest that Mayan daykeepers could have entertained a general framework by 453 CE or more generally after about three passes through a lunar table of 405 months. After another 3 × 405 months, 41 intereclipse intervals would match the table's intended stations, 30 of them three times, 20 four times, 12 five times, and 5 six times; spans of 94 and 399 months occurred seven times. It is plausible, therefore, that eclipse tables much like that of the Dresden Codex could have existed by around 550 CE. Given Schaefer's determination that partial solar eclipses with coverage under 50% are not noticeable if not anticipated (*3*), a hieroglyphic record of an eclipse with lower coverage of the solar disk would be evidence that a predictive eclipse table existed by that time.

Other intervals seem consequential for the eclipse table's design. Figure 3 shows that a span of 100 months occurs repeatedly between stations in successive odd and successive even month spans: 29, 129, and 229; 35 and 135; 41 and 141; 88 and 188; 94 and 194; 170 and 270; 176 and 276; 205 and 305; and 299 and 399. Additional examples occur between pairs of intervals, one or both of which occur only once: 47 and 147; 76 and 176; 111 and 211; 229 and 329; 252 and 352; 264 and 364; 270 and 370; and 305 and 405.

Across the eclipse table, however, an 88-month interval is far more consistent and consequential, the basis of an "eclipse-family construct" (*3*, *17*)—a series of eclipse-possible stations at 88-month intervals. All 55 intended stations of the eclipse table are captured by this construct, in three sets:

1) A set of 19 stations at months 59, 147, 235, 323, 6 (=323 + 88 − 405), 94, 182, 270, 358, 41, 129, 217, 305, 393, 76, 164, 252, and 340.

2) Eighteen at months 194, 282, 370, 53 (= 370 + 88 − 405), 141, 229, 317, 405, 88, 176, 264, 352, 35, 123, 211, 299, and 387.

3) Eighteen at months 329, 12 (= 329 + 88 − 405), 100, 188, 276, 364, 47, 135, 223, 311, 399, 82, 170, 258, 346, 29, and 117.

The pertinence of this interval could have been recognized very early; intereclipse intervals of 88 months and its multiples occur 195 times in the database. Not all attested intereclipse intervals would be among the eclipse-possible stations of the eclipse table; among those in the first 3 × 405 months, intervals of 17, 65, and 159 months have one attestation each, and 111 and 205 months each have two.

The dates of several eclipses in Fig. 2 suggest that recurrences might occur near the same placements in the Mayan 365-day year, and there are noteworthy relationships among nodal passages, new moons, and the 365-day calendar: The nearest within 405 months are at 19 × 365 = [40 × 173.30906] + 2.6376 days ≈ 235 months − 4.688 days; 28 × 365 = [59 × 173.30906] − 5.2345 days ≈ 346 months + 2.416 days; 9 × 365 = [91 × 173.30906] − 7.8721 days ≈ 111 months +7.105 days; and 1447 days = [4 × 365] − 13 days, an especially plausible Mesoamerican model for calendrical calculations.

Much more systematic, however, are recurrences in the Mayan divinatory calendar of 260 days. Teeple (*15*) observed that three nodal passages nearly equal 520 days: 3 × 173.30906 = [2 × 260] − 0.07282. As daykeepers were adepts in the divinatory calendar, they would have noticed near-recurrences of eclipse dates in it.

Figure 4 presents evidence for this expectation. Among eclipses in the first 3 × 405 months of Fig. 2 from 356 to 453 CE, 62 pairs are separated by intervals of at most 405 months; 60 correspond to stations of the eclipse table. Furthermore, these groups correspond to those

of the 88-month model described above: Those without a 177-day offset from a multiple of 520 days consist only of stations from the series 194, 282, 370, 53, 141, 229, 317, 405, 88, 176, 264, 352, 35, 123, 211, 299, and 387; those with 177 days added are from the series 59, 147, 235, 323, 6, 94, 182, 270, 358, 41, 129, 217, 305, 393, 76, 164, 252, and 340; and those with 177 days subtracted are from the series 329, 12, 100, 188, 276, 364, 47, 135, 223, 311, 399, 82, 170, 258, 346, 29, and 117 (underscores mark stations attested in Fig. 4). The agreement of these two structured sets might have fostered the 88-month model for intereclipse intervals in a 405-month table, even within this short a span.

Notably, every interval in Fig. 4 is offset by an even multiple of 260 days. Mayan daykeepers, being specialists in the 260-day calendar, must have recognized and used this in developing models to anticipate possible dates of solar eclipses. Applying these models, every interval in the Dresden table falls between 14 days before and 10 days after a multiple of 520 days or of such a multiple of ±177 days; among these is an interval of 6 months, averaging 177.184 days.

Figure 5 applies this model systematically. A noteworthy feature is its regular tripartite organization of intended and contrived stations: The 1st, 4th, and 7th complete segments begin and end with contrived stations that surround six intended stations; the next segments, 2, 5, and 8, consist exclusively of intended stations; and the third group, segments 3 and 6, are preceded but not followed by contrived stations.

Another regularity is that, for seven of the eight complete series of intended stations on which a solar eclipse might be observed, preceded and followed by contrived stations, the offset of a small number of days is constant for the first few stations and constant 1 day later for the rest of the series. The advance occurs no later than the station having no 177-day offset: twice at that interval ([3 × 520] + 5

Months	Average length (in days)	
**6**	= 177.184	= **0** × 260 + 177 + 0.184
**41**	= 1210.754	= **4** × 260 + 177 − 6.246
**94**	= 2775.875	= **10** × 260 + 177 − 1.125
111	= *3277.895*	= 12 × 260 + 177 − 19.105
**129**	= 3809.446	= **14** × 260 + 177 − 7.554
**164**	= 4843.016	= **18** × 260 + 177 − 13.984
**270**	= 7973.258	= **30** × 260 + 177 − 3.742
**305**	= 9006.829	= **34** × 260 + 177 − 10.171
**323**	= 9538.379	= **36** × 260 + 177 + 1.379
**340**	= 10040.399	= **38** × 260 + 177 − 16.601
**393**	= 11605.520	= **44** × 260 + 177 − 11.480

Months	Average length (in days)	
**29**	= 856.387	= **4** × 260 − 177 − 6.613
**47**	= 1387.938	= **6** × 260 − 177 + 4.938
**100**	= 2953.059	= **12** × 260 − 177 + 10.059
**135**	= 3986.629	= **16** × 260 − 177 + 3.629
**170**	= 5020.200	= **20** × 260 − 177 − 2.800
**188**	= 5551.750	= **22** × 260 − 177 + 8.750
205	= *6053.770*	= 24 × 260 − 177 − 9.230
**276**	= 8150.442	= **32** × 260 − 177 + 7.442
**346**	= 10217.583	= **40** × 260 − 177 − 5.417
**364**	= 10749.133	= **42** × 260 − 177 + 6.133

Months	Average length (in days)	
**35**	= 1033.571	= **4** × 260 − 6.429
**88**	= 2598.692	= **10** × 260 − 1.308
**141**	= 4163.813	= **16** × 260 + 3.813
**176**	= 5197.383	= **20** × 260 − 2.617
**194**	= 5728.934	= **22** × 260 + 8.934
**229**	= 6762.504	= **26** × 260 + 2.504
**299**	= 8829.645	= **34** × 260 − 10.355
**317**	= 9361.196	= **36** × 260 + 1.196
**352**	= 10394.766	= **40** × 260 − 5.234

Fig. 4. Frequency distribution of repeated intereclipse intervals ≤405 months among eclipses within 3 × 405 months of the first Mayan lunar records. Bolded month intervals are intended stations of the Dresden table; italicized intervals are not. Month 111, offset from [12 × 260] + 177 days, is a contrived station of the table; month 205, offset from [24 × 260] − 177 days, does not appear in the table and falls between its contrived stations at months 200 and 206.

Model		Station	Model		Station	Model		Station
**1×177**		**177**						
**2×177**		**354**						
3×520 − 6×177	***+ 4***	***502***	*10×520 − 4×177*	*− 3*	*4489*	***18×520 − 5×177***		***8475***
3×520 − 5×177	*+ 4*	*679*	*10×520 − 3×177*	*− 3*	*4666*	*18×520 − 4×177*		*8652*
**3×520 − 4×177**	**+**	**856**	10×520 − 2×177	− 3	4843	**18×520 − 3×177**		**8829**
**3×520 − 3×177**	**+ 4**	**1033**	**10×520 − 1×177**	**− 2**	**5021**	**18×520 − 2×177**	**+ 1**	**9007**
**3×520 − 2×177**	**+ 4**	**1211**	**10×520**	**− 2**	**5198**	**18×520 − 1×177**	**+ 1**	**9184**
**3×520 − 1×177**	**+ 4**	**1388**	10×520 + 1×177	− 2	5375	18×520	+ 1	9361
3×520	+ 5	1565	**10×520 + 2×177**	**− 2**	**5552**	**18×520 + 1×177**	**+ 1**	**9538**
**3×520 + 1×177**	**+ 5**	**1742**	**10×520 + 3×177**	**− 2**	**5729**	18×520 + 2×177	+ 1	9715
3×520 + 2×177	***+ 5***	***1919***	*10×520 + 4×177*	*− 2*	*5906*	*18×520 + 3×177*	*+ 1*	*9892*
3×520 + 3×177	*+ 5*	*2096*	*10×520 + 5×177*	*− 2*	*6083*	**20×520 − 2×177**	**− 6**	**10,040**
**5×520 − 2×177**	**− 2**	**2244**	13×520 − 3×177	+ 2	6231	**20×520 − 1×177**	**− 6**	**10,217**
5×520 − 1×177	− 1	2422	13×520 − 2×177	+ 3	6409	20×520	− 5	10,395
5×520	− 1	2599	13×520 − 1×177	+ 3	6586	**20×520 + 1×177**	**− 5**	**10,572**
**5×520 + 1×177**	**− 1**	**2776**	**13×520**	**+ 3**	**6763**	**20×520 + 2×177**	**− 5**	**10,749**
5×520 + 2×177	− 1	2953	**13×520 + 1×177**	**+ 3**	**6940**	**20×520 + 3×177**	**− 5**	**10,926**
**5×520 + 3×177**	**− 1**	**3130**	13×520 + 2×177	+ 3	7117	**20×520 + 4×177**	**− 5**	**11,103**
8×520 − 5×177	*+ 3*	*3278*	*15×520 − 3×177*	*− 4*	*7265*	*23×520 − 4×177*	*− 1*	*11,251*
8×520 − 4×177	+ 3	3455	**15×520 − 2×177**	**− 4**	**7442**	23×520 − 3×177	− 1	11,428
8×520 − 3×177	+ 3	3632	**15×520 − 1×177**	**− 4**	**7619**	**23×520 − 2×177**	**− 1**	**11,605**
**8×520 − 2×177**	**+ 3**	**3809**	**15×520**	**− 4**	**7796**	**23×520 − 1×177**	**− 1**	**11,782**
**8×520 − 1×177**	**+ 4**	**3987**	**15×520 + 1×177**	**− 4**	**7973**	**23×520**	**− 1**	**11,959**
**8×520**	**+ 4**	**4164**	15×520 + 2×177	− 4	8150			
8×520 + 1×177	+ 4	4341	15×520 + 3×177	− 4	8327			

Fig. 5. Eclipse station model spanning 405 months. Generalized from intereclipse intervals among eclipses observable in the Mayan territory from the first 1215 months (3 table lengths) of solar eclipses following the sunrise eclipse of 27 June 363 CE, matching every station of the Dresden table. Solid horizontal lines divide groups defined by different multiples of 520 days; dashed lines separate one or two contrived stations from preceding or following intended stations at the same multiple of 520 days. Stations at intervals between pairs of eclipses visible in the Mayan territory from 373 to 471 CE are in bold type, with the larger type for intervals instantiated by three or more pairs; contrived stations are in italics. Every station in the model turns out to fall on the exact day specified in the eclipse table (compare Fig. 1); actual solar eclipses can differ from the Dresden stations by 1 day.

and [20 × 520] − 5) in the first and last complete series; one station before in the second, third, and fourth series ([5 × 520] − 1, [8 × 520] + 4, and [10 × 520] − 2); and two stations before in the fifth and seventh series ([13 × 520] + 3 and [18 × 520] + 1). Each shift is the occasion of a 178-day interval in the table. In the sixth series, among the stations around 15 × 520 days, the offset is constant throughout.

Each contrived station in this model is part of an uninterrupted series of increasing multiples of 177 days in sequence with either the immediately preceding or immediately following intended stations at the same multiple of 520 days. When two contrived stations occur in sequence, each is offset from the same multiple of 520 days, part of the same component of the model.

The visual organization of the eclipse table turns out to express this organization of the stations. In Fig. 5, a solid rectangle encloses every complete sequence of intended stations; in Fig. 1, a dashed first station is placed immediately before a captioned image (see Fig. 6). (Each image depicts a supernatural figure or an eclipse glyph, below a rectangular feature that Mayanists refer to as a “sky band.”)

The more typical length of an eclipse table would have been 11,960 rather than 11,959 days (*2*). In the Monte Alban survey of lunar first appearances (table S2), 81.627% are 11,960 and 14.607% are 11,959 days long (3.766% are 11,961 days long), and 11,960 days is the length of 44 or 45 of 49 1447-day models depending on the starting sequence, with the rest being 11,959 days. Tables of these lengths would have been the source of most daykeepers’ experiences. Under the calibration model discussed above, an 11,960-day table would be implemented by including a 178-day group in the set at [15 × 520] ± 177*k* − 4, the only complete set that lacks such a group in the 11,959-day table; it would be implemented in the model by adding 1 day to the last few offsets in that series. Given the regularities in the rest of the table, it would have had one of only two possible placements: In no other sequence of intended stations does the offset begin at the first station, and in none does it follow a station with no 177-day offsets. Therefore, the missing 178-day interval would almost surely have been placed at [15 × 520] − [1 × 177] − 3 or at [15 × 520] − 3 days, i.e., day 7620 or 7797. Thereafter, barring further adjustments, the stations of the 11,960-day table would fall 1 day later than in an 11,959-day table, starting at day 7620 or 7797.

Figure 7 presents such a table with the shift placed, arbitrarily, at day 7797. Offsets would range from −5 to +5 days, as in all frequently recurring intervals among eclipses observable between 363 and 490 CE—whereas, in the 11,959-day table, the offsets at days 10,040 and 10,217 are −6 days, an overall range of −6 to +5 days. The final series of offsets from [23 × 520] − 177*k* days would be followed in principle, after month 405 at 23 × 520 days, by stations at [23 × 520] + 177 and [23 × 520] + [2 × 177]; this is effectively the same model as that of the initial stations at [0 × 520] + 177 and [0 × 520] + [2 × 177]. This elaborated series partially parallels those of groups 3 and 6, beginning with a single contrived station, ending with an intended station, and preceding a series that begins with a pair of contrived stations.

Another likely departure is the placement of the 178-day station in the seventh group, the 18 × 520-day series. In the 11,959-day Dresden table, the 178-day interval occurs so early only in this group at the second station, two stations before the one with no 177-day offset. It seems likely that it was shifted to this early placement because there was no 1-day increment in the preceding set of intended stations. If so, this increment was likely placed at the third intended station in both the 15 × 520 and 18 × 520 groups of an 11,960-day table.

The earliest implementation of models based on multiples of 520 days might have used unvarying offsets for each series of six or seven successive intended stations; however, in daykeepers’ early experience, intervals of 6 months would vary from 176 to 178 days. In the Monte Alban survey (table S1, spans in days for each interval of 1 to 405 months), the frequencies are 18.145% of 176, 46.955% of 177, 33.284% of 178, and 1.616% of 179 days. The 1447-day model is more constrained: Every 6-month sequence consists either of [3 × 30] + [3 × 29] = 177 days or of [4 × 30] + [2 × 29] = 178 days; a 30-month sequence, averaging 885.9177 days, would almost always equal [2 × 502] + 59 = 886 = [4 × 177] + 178 days but rarely 502 + 443 − 60 = 885 = 5 × 177 days (1 to 3 of 49 starting points; 2.53 on average); and a 36-month sequence would usually be [2 × 5 02] + 59 = 1063 = [5 × 177] + 178 days but rarely [2 × 502] + 60 =

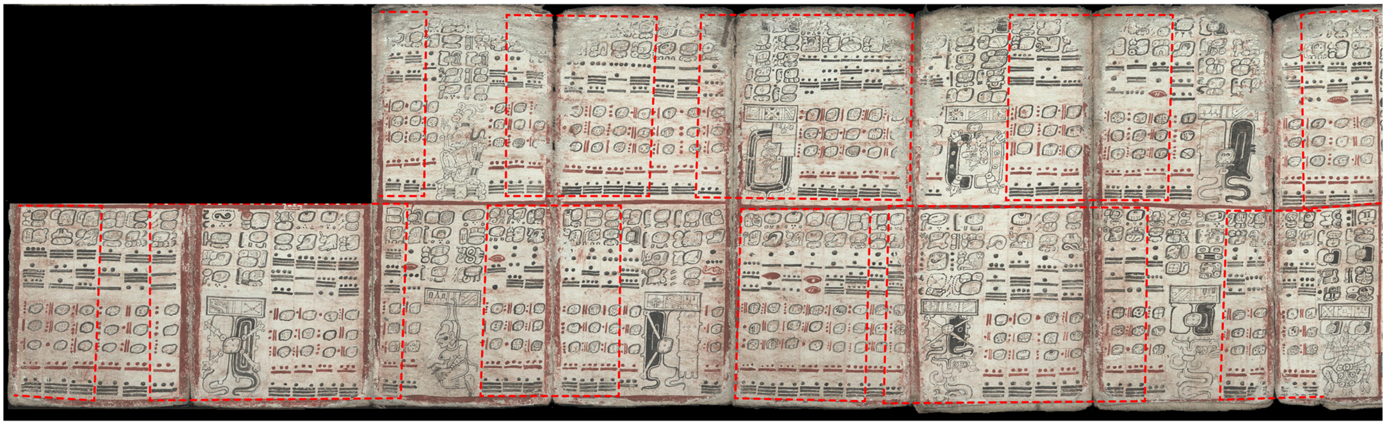

Fig. 6. Eclipse table of the Dresden Codex. The dashed boxes surround the sequences of six or (once) seven stations categorized as “intended” in Fig. 1; stations between successive dashed boxes are contrived stations. The first two stations, at the upper left, lack a vertical dashed line to the left because four intended stations would precede the table’s first eclipse station; the last four stations, at the lower right, lack a vertical dashed line to the right because two intended stations would follow them. Dashed lines form rectangles surrounding each series of consecutive intended stations; a picture, above noncalendrical glyphic passages that contain no numerals, usually occurs between successive rectangles and otherwise immediately before the last calendrical column in a rectangle. Adapted from Justeson *(3)*.

Model		Station	Model		Station	Model		Station
1×177		177						
2×177		354						
3×520 – 6×177	+	502	10×520 – 4×177	–	4489	18×520 – 5×177	+	8476
3×520 – 5×177	+	679	10×520 – 3×177	–	4666	18×520 – 4×177	+	8653
3×520 – 4×177	+	856	10×520 – 2×177	–	4843	18×520 – 3×177	+	8830
3×520 – 3×177	+	1033	10×520 – 1×177	–	5021	18×520 – 2×177	+	9008
3×520 – 2×177	+	1211	10×520	–	5198	18×520 – 1×177	+	9185
3×520 –1×177	+	1388	10×520 + 1×177	–	5375	18×520	+	9362
3×520	+	1565	10×520 + 2×177	–	5552	18×520 + 1×177	+	9539
3×520 + 1×177	+	1742	10×520 + 3×177	–	5729	18×520 + 2×177	+	9716
3×520 + 2×177	+	1919	10×520 + 4×177	–	5906	18×520 + 3×177	+	9893
3×520 + 3×177	+	2096	10×520 + 5×177	–	6083	20×520 – 2×177	–	10,041
5×520 – 2×177	–	2244	13×520 – 3×177	+	6231	20×520 – 1×177	–	10,218
5×520 – 1×177	–	2422	13×520 – 2×177	+	6409	20×520	–	10,394
5×520	–	2599	13×520 – 1×177	+	6586	20×520 + 1×177	–	10,571
5×520 + 1×177	–	2776	13×520	+	6763	20×520 + 2×177	–	10,748
5×520 + 2×177	–	2953	13×520 + 1×177	+	6940	20×520 + 3×177	–	10,925
5×520 + 3×177	–	3130	13×520 + 2×177	+	7117	20×520 + 4×177	–	11,102
8×520 – 5×177	+	3278	15×520 – 3×177	–	7265	23×520 – 4×177		11,252
8×520 – 4×177	+	3455	15×520 – 2×177	–	7442	23×520 – 3×177		11,429
8×520 – 3×177	+	3632	15×520 – 1×177	–	7619	23×520 – 2×177		11,606
8×520 – 2×177	+	3809	15×520	–	7797	23×520 – 1×177		11,783
8×520 – 1×177	+	3987	15×520 + 1×177	–	7974	23×520		11,960
8×520	+	4164	15×520 + 2×177	–	8151			
8×520 + 1×177	+	4341	15×520 + 3×177	–	8328			

Fig. 7. Suggested Mayan model for an 11,960-day eclipse table.

1064 = [4 × 177] + [2 × 178] (3 to 5 of 49 starting points; 3.776 on average). This would suggest that every sequence of six 6-month intervals would include one of 178 days; the lack of such a sequence from station 7442 to 8237 in the Dresden table was evidently designed to shorten its length to 11,959 days from a standard table of 11,960 days.

Lounsbury (*2*) demonstrated that the Dresden table was composed as such a revision, showing from patterns of scribal errors that it was partly copied from a prior table. For each station, one line of intervals gives the number of days by which it follows the prior station, and another gives the number of days by which it follows the table's base date. He observed that "the very first number of the series of cumulative totals ... is written as 7.17" (Mayanist notation for [7 × 20] + 17 = 157 days). "But obviously it should be 8.17" [177 days] "since the first cumulative total of the series must necessarily be the same as the first contribution to the total, which is written below as it should be, as 8.17, in the series of eclipse half-year intervals.... It is clear that one dot was omitted in the uinal or 'scores' position by the copyist... Another copying error on the same page of the codex is in the fourth cumulative total, written as 1.15.14; but this should be 1.15.19. It is obvious that one bar was omitted in the kin or 'units' position by the copyist, resulting in an error of 5 days." He also argued that the existing table, at 11,959 days long, therefore shifted from a base on day 169 of the divinatory calendar to a final date on day 168, which he concluded was to be the "new base" of the next several tables (*2*)—this was based on the assumption that the base of the new table would be the final day of the current table.

Every station of the eclipse table exactly matches the corresponding station in the formal model of Fig. 5; they must have been calculated, as records of many observed eclipses would have a discrepancy of ±1 day. The distance between actual eclipses at the table's base date and 405 months later happens to have been 11,960 days, not 11,959 days, at the 1043, 1076, 1083, and 1116 CE candidates for the table's base date—both in early afternoon from the 1083 base and the others from afternoon at the base to early morning at month 405. This is expected: The frequency of this length in the Monte Alban corpus is 81.627% (table S1), so the probability that the spans for all four base dates chance to be 11,960 days is about 44.163%.

Willson (*18*) was the first scholar to realize that pages 51a to 58b of the Dresden Codex were an eclipse table. Teeple (*15*) elaborated that its stations yield coincidences "so many and so remarkable that it must surely be intended for a table of eclipse syzygies, the only possible alternative being to correlate the moons with the tzolkin days." Figures 5 and 7 provide evidence for a more precise version of the second alternative: that eclipse prediction, and the table, emerged by correlating dates of observed solar eclipses with the 260-day calendar.

Composing a successor eclipse table

Previous treatments have assumed that the base of a new table would be set on a current table's final date, normally day 11,960, of a current table. However, a station at day 11,960 averages 1.6749 days later than the base relative to the nodes; if the next table began on that day, new prenodal stations should have been introduced on days 2067 (then 11.145 days prenodal) and 6054 (10.254 days prenodal), which do not appear in the table. The interpretation of several stations would change. Three contrived stations would become eclipse-possible stations: Days 4666, 8653, and 11,252 would become the extreme prenodal station in their set, immediately before the previous table's prenodal extremes at days 4843, 8830, and 11,429. Four intended stations would become contrived, days 3130, 7117, 9716, and 11,103 of the new table exceeding the postnodal limit of 11.775 days. A consequence of such interpretive changes is that the pattern of intervals among the stations would not reliably distinguish eclipse-possible dates from contrived dates, conflicting with the pattern of 502-day versus 503-day intervals as a design feature of the table.

On the first repetition of a table, the probability that an unanticipated eclipse would occur at an abnodal extreme is relatively low, as observable eclipses are rarest there; after a second shift, the final station in almost every set would fall beyond postnodal limits, and almost every contrived station 6 months before the first intended station in a series would fall within prenodal limits.

These features would be exacerbated on each repetition. In the next 405 months, another contrived station, at day 9893, would have to be replaced by a station 11.490 days prenodal on day 9864; the introduced stations at days 2067 and 6054 would shift 1.675 days closer to a node; and the sole or final station in the other six sets of contrived stations (on days 679, 3278, 4666, 7265, 8653, and 11,252) would all fall within the prenodal range of visibility.

The intervals at 223 and 358 months turn out to be key to successive revisions of the table. These stations have the smallest average abnodal deviations within 405 months, postnodal by 0.0982 days for the station at 358 months and prenodal for the station at 223 months by 0.4229 days, about 4.3 times as long. From today's perspective, on the basis of abnodal data, the base of an upcoming table that had begun during the applicability of a current table would have been best placed at one of these two stations; in the long term, a mix of the two would be necessary.

Without an explicit concept of eclipse nodes, these ratios would not have been part of daykeepers' approach to eclipse prediction. Commensuration with the 260-day calendar would not be helpful (see Fig. 8). Successive improving sets of ratios of months to multiples of 260 days are 9:1, 44:5, and 361:41, but no odd multiple of 260 days is a station in the table.

The 358-month interval was likely the first to be recognized by Mayan calendar specialists as key to a procedure for resetting eclipse tables: For daykeepers, it is the only station exactly at the middle of a series of intended stations (see Fig. 6)—the fourth of seven stations—while every other series consists of only six stations. From our perspective, it is the station closest to a node. Furthermore, a series of seven solar eclipse stations viewed from a particular location requires very close to the maximum of 13 hours and 3 min between sunrise and sunset—the first eclipse in the series occurring during or very near sunrise and the last during or very near sunset.

Nonetheless, repeated resettings at month 358 would eventually yield a base too far from a node for a reliable table. The first interval in Fig. 8 to exceed the length of the eclipse table, 1655 months, emerges as key to long-term resettings, with an average prenodal shift under 51 min. Spanning nearly 134 years, it would involve the accumulating activities and observations of generations of daykeepers. Among the 145 eclipses of Fig. 2, only 51 are not at a multiple of 1655 months from any other. There is a set of five at various multiples of 1655 months from one another and eight sets of three at such multiples; 33 other pairs occur at some multiple apart, 20 at exactly 1655 months. Overall, 95 (65.5%) of these 145 eclipses are separated by some multiple of 1655 months from another, the highest proportion among Fig. 8's intervals. Since 1655 months = [4 × 358] + 223 months, a 223-month adjustment corrects for accumulating deviations from resettings of the table's base at month 358.

The successive improvements of the month spans of Fig. 8 are consistent with what daykeepers would have observed for histories of each span's multiples, as follows:

1) Spans of 1 × 88 through 26 × 88 months are all attested, persisting longer than any shorter span; its double, 176 months, is attested 28 times, more often than any shorter span. However, its performance declines, roughly linearly (r^2 = 0.8217), through its 19th multiple, about 135 years, with just one instance. This is the shortest interval to survive past the first four 405-month spans. After its 12th multiple, an interval of $88n + 1$ month appears, becomes common, and continues far longer—through [88 × 48] + 1 months, about 341$^1/_2$ years.

2) The span of 223 months has much greater longevity; excluding its outstandingly common multiple at 669 months, the first nine multiples (1, 2, and 4 to 10) would average 13.44 instances, the next nine (11 to 19) average 9.33, and the final nine (20 to 28) average only 2.67—a span of nearly 505 years. None occurs among the remaining multiples (29 to 43).

3) Multiples of 358 months stand out among the eclipses observable in the Mayan territory from 356 to 1148 CE; all multiples between 1 × 358 and 24 × 358 months are attested, along with one more recurrence among its last multiples (25 to 27) in the 784 years of the survey.

4) Six multiples of 1655 months fit in the 784 years of the sample, all six attested.

n (nodal passages)	Julian years	Months	*n* × ratio	Abnodal deviations (in days)	Attestations among 146 eclipses
1	0.485	6	5.8688	3.87446	5.52% of 145
7	3.315	41	41.0816	−2.40939	4.86% of 144
8	3.8	47	46.9504	1.46507	12.5% of 144
15	7.115	88	88.032	−0.94431	13.19% of 144
23	10.915	135	134.9824	0.52076	11.27% of 142
38	18.03	223	223.0143	−0.42356	5.67% of 141
61	28.944	358	357.9967	0.0972	7.09% of 141
282	133.807	1655	1655.0012	−0.03476	16.13% of 124

Fig. 8. Successive best ratios of nodal passages to lunar months.

Reconstructing table histories

In the 260-day divinatory calendar, the eclipse table's base is day 169 (13 Muluc), and its final station is day 168 (12 Lamat). Four solar eclipses of Fig. 2 were observable in the Mayan territory within a day of one of them: day 168 on 9 June 1043 and 7 March 1076 and day 167 on 19 April 1083 and 16 January 1116. Four placements of the table are consistent with these dates: from 1043 to 1076; 1076 to 1108; 1083 to 1116; or 1116 to 1148. These date ranges indicate that the table's model originated in northern Yucatan.

Figure 9 presents hypothetical histories for these four candidate bases in chronological order. For each candidate history, every eclipse visible in Yucatan over 405 months would have fallen on an intended station of the eclipse table.

If the Dresden table's base was at 1043, 1083, or 1116 CE, the table would have both begun and ended with a solar eclipse visible in the Mayan territory. Historically, this notable feature occurred so rarely that it seems the likely basis for enshrining this particular table, at its specific place in the divinatory calendar, in the Dresden Codex. For each placement, a prior history would be consistent for all five placements of the 223-month adjustment and would have had histories consistent from at least 361 CE, the time of the earliest known Mayan lunar day count. From each of these bases, there are five alternative histories depending on when a new table began after 223 months rather than 358 months, and for each pair of these alternatives, three of every five successive tables have identical date ranges. For each base, the alternative with the most stations among its candidate histories is presented in Fig. 9.

In a table beginning with the 1043 or 1116 base, one earlier predecessor table would also have begun and ended with an observed solar eclipse: for the 1043 base, with a table base in 797 CE, and for the 1116 base, with a table base in 790 CE. A table with a base in 1083 had no prior instance of an eclipse at both its first and last stations.

If the table's base fell in 1076, it was set at month 223 of its predecessor; set at month 358, local eclipses in the predecessor would have fallen on month 18, which is not a station in the eclipse table. An ancestral table set in 790 CE would have begun and ended with a local eclipse; the base of the Dresden table would have instantiated one but not month 405. This placement seems the least promising candidate for the eclipse table.

An independent line of evidence suggests that a 1043 CE base is implausible. As noted above, eclipses at abnodal extremes are rare compared to those nearer the nodes. In the data presented in Fig. 9, a linear equation predicts station frequency from the station's absolute abnodal distance for bases at 1076 (r^2 = 0.5585), 1083 (r^2 = 0.4328), and 1116 CE (r^2 = 0.5278); for the 1043 CE base, there is no correlation (r^2 = 0.0022). The most likely placements for the table appear to be 1083, originally proposed by Makemson (*19*) and

	374 May 27	392 Jun 7	421 May 17	450 Apr 27	479 Apr 8	508 Mar 17	526 Feb 28	555 Mar 8	584 Feb 17	613 Jan 26	642 Jan 6	660 Jan 17	688 Dec 28	717 Dec 7	746 Nov 17	775 Oct 29	793 Nov 8	822 Oct 18	851 Sep 28	880 Sep 8	909 Aug 18	927 Aug 29	956 Aug 8	985 Jul 20	1014 Jun 30	1403 Jun 9
**Prior table's station as base**		223	358	358	358	358	223	358	358	358	358	223	358	358	358	358	223	358	358	358	358	223	358	358	358	358
	270	47	41	**0**	141	35	**0**	41	6	76	6	35	12	129	41	35	**0**	6	**0**	12	182	47	94	**0**	94	**0**
	276	53	88	6	176	88	53	59	47	123	100	53	88	229	47	94	47	41	6	47	270	82	141	129	135	65
	305	82	188	35	264	223	94	123	106	141	129	370	94	264	141	182	53	76	47	147	305	176	311	147	264	82
	370	147	223	305	282	276	141	170	305	217	258		176	317	235	223	129	94	53	235	399		358	217	358	229
		241	229		346	317	323	276		229	276		223	323	299	270	147	182	100	264				305		241
		258	317		393	364	352	311		264			264	399	311	276	264	311	317	311				323		276
		352	358				399	317		282			282	**405**	393	352	323	358	352	323						**405**
		399	364					364		317			346			370	364	364	370							
			393					**405**		364							399	**405**	**405**							

	360 Mar 3	389 Feb 12	407 Feb 23	436 Feb 3	465 Jan 13	493 Dec 24	522 Dec 3	540 Dec 14	569 Nov 24	598 Nov 4	627 Oct 14	656 Sep 23	674 Oct 5	703 Sep 15	723 Aug 25	761 Aug 4	790 Jul 16	808 Jul 26	837 Jul 6	866 Jun 16	895 May 27	924 May 6	942 May 17	971 Apr 27	1000 Apr 7	1029 Mar 17	1058 Feb 25	1076 Mar 7
**Prior table's station as base**		358	223	358	358	358	358	223	358	358	358	358	223	358	358	358	358	223	358	358	358	358	223	358	358	358	358	223
	41	88	59	6	123	82	41	141	94	123	35	76	188	41	47	53	**0**	82	**0**	135	53	**0**	270	129	35	82	47	**0**
	53	94	76	41	317	100	94	170	129	252	47	94	264	82	82	117	41	141	129	170	82	88	317	176	123	176	59	12
	317	123	170	47	352	164	135	217	135	299	82		270	100	135	129	88	182	176	188	129	123		305	141	241	94	41
		188	217	135		211	182	235	182	317	100		352	164	141	211	94	217	182	223	141	217		323	270	258	223	88
		282	264	176		264	364	299	223	393	135		399	305	217	270	170	252	223	323	358			393	311	**405**	235	176
		299	364	182		399	393	346	282	405	182			**405**	223	358	188	270	229								264	229
		393	399	211							276				317	399	305	358	276								311	264
			**405**								305						364										399	329
																	**405**											364

	367 Apr 15	385 Apr 25	414 Apr 5	443 Mar 17	427 Feb 24	501 Feb 3	519 Feb 15	548 Jan 26	577 Jan 4	605 Dec 15	634 Nov 26	652 Dec 6	681 Nov 15	710 Oct 26	739 Oct 7	768 Sep 16	786 Sep 26	815 Sep 6	844 Aug 17	873 Jul 27	902 Jul 7	920 Jul 18	949 Jun 28	978 Jun 8	1007 May 19	1036 Apr 28	1054 May 10	1083 Apr 19
**Prior table's station as base**		223	358	358	358	358	223	358	358	358	358	223	358	358	358	358	223	358	358	358	358	223	358	358	358	358	223	358
	229	6	82	47	35	12	88	53	6	35	12	123	100	12	47	29	47	53	41	47	41	47	182	41	35	88	94	**0**
	358	135	129	88	229	76	141	82	41	164	47	141	176	76	53	41	88	94	88	82	53	135	229	88	53	153	106	88
	364	141	176	94	264	123	182	129	47	211	94		182	217	129	123	135	129	94	100	270	170	399	217	182	170	141	141
	393	170	276	123	352	176	229	147	94	229	188		264	317	135	182	141	164	135	135	358	264		235	223	317	270	176
		235	311	393	370	311		211	135	305	217		311	352	229	270	217	182	141	235	393			305	352	329	282	241
		329	317			364		258	194	317	346		352	**405**	323	311	235	270	188	323				393		364	311	276
		346	**405**			**405**		364	393	352	364		370		387	358	352	399	**405**	352							358	**405**
								399		370					399	364				399								
								**405**		**405**																		

	371 Feb 2	400 Jan 12	428 Dec 22	447 Jan 2	475 Dec 14	504 Nov 22	533 Nov 2	562 Oct 13	580 Oct 24	609 Oct 3	638 Sep 13	667 Aug 25	696 Aug 3	714 Aug 14	743 Jul 25	772 Jul 5	801 Jun 15	830 May 25	848 Jun 4	877 May 16	906 Apr 26	935 Apr 5	964 Mar 16	982 Mar 27	1011 Mar 7	1040 Feb 14	1069 Jan 25	1098 Jan 5	1116 Jan 16
**Prior table's station as base**		358	358	223	358	358	358	358	223	358	358	358	358	223	358	358	358	358	223	358	358	358	358	223	358	358	358	358	223
	182	53	94	**0**	182	29	**0**	29	**0**	117	**0**	276	**0**	29	**0**	76	35	**0**	41	**0**	6	82	**0**	41	6	41	88	59	**0**
	311	147	129	41	217	76	47	76	47	164	47	352	82	170	6	135	53	88	47	35	223	358	47	170	135	106	100	94	88
	317	164	135	47	305	129	229	182	88	182	141	358	129	270	82	223	170	217	88	53	311	**405**	217	188	176	123	129	223	123
	346	258	223	76	323	264	258	217	147	258	170		170	305	88	264	229	264	94	88	346		264	258	305	270	176	311	176
		305	264	346	387	317	305	223	346	270	299		188	358	182	311	270	270	141	188			393	346	399	282	264	346	188
		352	270			358	323	270		305			252	364	276	317	305	311	358	276				364		317	317	399	264
						**405**	387	311		323			393		340	393	340	317	393	305							352		299
								370		358					352		358	364		352									**405**
										**405**										364									

Fig. 9. Four hypothetical eclipse table histories, projected backward from the Dresden Codex eclipse table base at 1043, 1076, 1083, and 1116 CE.

championed by Lounsbury (*2*), or 1116 CE, which Teeple (*15*) discussed as the midpoint of his suggested range of possible dates (1066 to 1165 CE). Justeson had previously argued for a 1083 base (*3*).

One other placement of the table, from 1108 to 1148, would have had appropriate placements of eclipse stations. However, the history of such a table could go back no earlier than 808 CE, because the last prior table would have had an eclipse at month 70 or 205—neither being a station in the eclipse table. Daykeepers would have had sufficient evidence for the table's stations long before that date: From the first Mayan lunar day records until 808 CE, 51 of the 55 stations had corresponded to between 4 and 17 intereclipse attestations; among the rest, month 117 had none, month 376 had one, and

months 12 and 29 had two. Furthermore, unlike the other candidate placements, no eclipse would have been observable at either its base or at month 405. It seems unlikely that 1108 CE could have been a placement of the eclipse table.

Concluding remarks

This paper has provided evidence for developments of lunar theory among Mayan calendar specialists from about 350 CE. It focuses on the methods they used to anticipate the dates on which solar eclipses might occur in their territories, as reflected in an eclipse table in the Dresden Codex, most likely covering eclipse-possible dates during a 32¾-year period beginning in either 1083 or 1116 CE.

A long-standing assumption that the table was restarted at its final position is rejected here. Given that assumption, after a single pass through the table, a very few of the successor table's eclipse-possible stations would fall outside the intervals during which the Moon would partially obscure the Sun, and the Moon would partially obscure the Sun on very few positions that were not recorded in the successor table. In place of this assumption, this paper proposes that the first date of a new table would usually be set at the 358th month of a current table; this station has the smallest deviation from a precise alignment of the Sun and Moon, only about 2 hours and 20 min earlier than in the previous alignment. This procedure would also entail that, occasionally, the first date in a successor table would be set at the 223rd month, about 10 hours and 10 min later relative to that alignment, to adjust for the gradually accumulating deviations of resettings at month 358. Such revisions would maintain the viability of the table indefinitely, with departures of under 51 min over 134 years.

Unexpectedly, on the basis of an earlier scholarship, the earliest version of the eclipse table seems to have been a repurposed revision of a less complex table, which listed 405 successive lunar months, each lasting either 29 or 30 days. If the placements of such a table were calculated by equating 49 lunar months with 1447 days, 405 months would have been the first entry to be a multiple of 260 days. This suggests that the 405-month eclipse table had emerged from a lunar calendar in which the 260-day divinatory calendar commensurated the lunar cycle.

Supplementary Materials

The PDF file includes:
Supplementary Text
Legends for tables S1 and S2

Other Supplementary Material for this manuscript includes the following:
Tables S1 and S2

REFERENCES AND NOTES

1. J. M. Steele, *Observations and Predictions of Eclipse Times by Early Astronomers* (Springer, 2000).
2. F. G. Lounsbury, "Maya numeration, computation, and calendrical arithmetic" in *Dictionary of Scientific Biography* (Scribner, 1978), vol. 17, pp. 759–818.
3. J. Justeson, A cyclic-time model for the structure of the eclipse table of the Dresden Codex. *Ancient Mesoam.* **28**, 507–541 (2017).
4. United States Naval Observatory, Astronomical Applications Department, "Table of sunrise/sunset, moonrise/moonset, or twilight times for an entire year," accessed for the years 1700 and 2100 at Calakmul, Campeche, Mexico (day lengths at solstices do not shift by more than a few seconds over two thousand years); https://aa.usno.navy.mil/data/RS_OneYear.
5. X. Jubier, "Five Millennium (−1999 to +3000) Canon of Solar Eclipses Database"; http://xjubier.free.fr/en/site_pages/solar_eclipses/5MCSE/xSE_2_Five_Millennium_Canon.html.
6. I. Cases, A. Lacadena, "Operación III.5. Estudios Epigráficos, Temporada 2014" [Operation III.5. Epigraphic Studies, Season 2014], in *Proyecto Petén-Norte Naachtun 2010-2014: Informe final de la Quinta Temporada de Campo 2014*, P. Nondédéo, J. Hiquet, D. Michelet, J. Sion, L. Garrido, Eds. (Instituto de Antropología e Historia, 2015), pp. 371–384.
7. P. Nondédéo, A. Lacadena, and I. Cases, Teotihuacanos y mayas en la «entrada» de 11 Eb' (378 dC): Nuevos datos de Naachtun, Petén, Guatemala [Teotihuacans and Mayas at the «entrada» of 11 Jan' (378 AD): New data from Naachtun, Petén, Guatemala]. *Rev. Esp. Antropol. Am.* **49**, 53–75 (2019).
8. D. Stuart, "'The arrival of strangers': Teotihuacan and Tollan in Classic Maya history" in *Mesoamerica's Classic Heritage: From Teotihuacan to the Toltecs*, D. Carrasco, L. Jones, S. Sessions, Eds. (University Press of Colorado, 2000), pp. 467–490.
9. C. A. Pool, "Strangers in a strange land: ethnicity and ideology at an enclave community in Middle Classic Mesoamerica" in *Ancient Images, Ancient Thought: the Archeology of Ideology*, A. Sean Goldsmith, S. Garvie, D. Selin, J. Smith, Eds. (University of Calgary Press, 1992), pp. 41–55.
10. D. Kelley, E. Milone, *Exploring Ancient Skies: An Encyclopedic Survey of Archaeoastronomy*. (Springer, 2005).
11. F. Espenak, J. Meeus, "Five millennium canon of solar eclipses: −1999 to +3000 (2000 BCE to 3000 CE)" (Technical Paper 2006-214141, National Aeronautics and Space Administration, 2006).
12. W. Chafe, J. Justeson, Floyd Glenn Lounsbury. *Language* **75**, 536–566 (1999).
13. L. Satterthwaite Jr, *Concepts and Structures of Maya Calendrical Arithmetic* (Museum of the University of Pennsylvania, The Philadelphia Anthropological Society, no. 3., University Museum, 1947).
14. A. Belenkiy, E. Vila Echagüe, History of one defeat: Reform of the Julian calendar as envisioned by Isaac Newton. *Notes Rec. R. Soc.* **59**, 223–254 (2005).
15. J. Teeple, *Maya Astronomy*, Contributions to American Archaeology, vol. I, no. 2, Carnegie Publications in Anthropology no. 403 (Carnegie Institution of Washington, 1930).
16. J.-I. Cases, J.-A. Belmonte, A. Lacadena, "Análisis de uniformidad de las Series Lunares mayas del periodo Clásico: Primeros resultados" [Uniformity analysis of the Classic Period Mayan Lunar Series: Initial results] in *Etno y arqueoastronomía en las Américas: Memorias del Simposio ARQ-13 del 51° Congreso Internacional de Americanistas*, M. Boccas, J. Broda, G. Pereira, Eds. (Universidad de Chile, 2004), pp. 195–210.
17. J. Justeson, "Modelling indigenous Mesoamerican eclipse theory" in *Cosmology, Calendars, and Horizon-Based Astronomy in Ancient Mesoamerica*, A. Dowd, S. Milbrath, Eds. (University Press of Colorado, 2015), pp. 301–349.
18. R. M. Willson, *Astronomical Notes on the Maya Codices* (Peabody Museum Press, 1924), vol. 6, no. 3, 74 pp.
19. M. W. Makemson, *The Astronomical Tables of the Maya*, Carnegie Institution of Washington Publications, no. 546, Contributions to American Anthropology and History no. 42 (Carnegie Institution of Washington, 1943), pp. 183–221.

Acknowledgments: This work builds most directly on the work of F. Lounsbury (d. 1998) and on the publications and advice of A. Aveni, with both of whom J.J. has had many helpful interactions spanning several decades. **Funding:** We have received no funding in support of this research. **Author contributions:** J.J. is responsible for the text; J.J. and J.L. are jointly responsible for copyediting. Figures were produced by J.L. from data tables by J.J. For the Supplemental Materials, J.L. produced a database (table S2) of first appearances of the lunar crescent at Monte Alban from 650 to 0 BCE, from which J.J. produced a database (table S1) of percentages of spans in days for each span in months, from 1 to 405 months. J.L. was responsible for interfacing with *Science Advances* concerning this paper. **Competing interests:** The authors declare that they have no competing interests. **Data and materials availability:** All data needed to evaluate the conclusions in the paper are present in the paper and/or the Supplementary Materials.

Submitted 15 October 2024
Accepted 26 September 2025
Published 22 October 2025
10.1126/sciadv.adt9039
